# Immune Phenotypic Characterization of a TRAIL-Knockout Mouse

**DOI:** 10.3390/cancers15051475

**Published:** 2023-02-25

**Authors:** Ani K. Stoyanova, Arne Sattler, Elisabeth M. Hahn, Nina A. Hering, Marco Arndt, Johannes Christian Lauscher, Fiona Speichinger-Hillenberg, Katja Kotsch, Ann-Kathrin Berg, Katharina Beyer

**Affiliations:** Department of General, Visceral and Vascular Surgery, Campus Benjamin Franklin, Charité-Universitätsmedizin Berlin, Hindenburgdamm 30, 12203 Berlin, Germany

**Keywords:** TRAIL: immunological phenotype, lymphocytes, dendritic cells, knockout mouse

## Abstract

**Simple Summary:**

The role of the TNF-superfamily member TRAIL (TNF-related apoptosis inducing ligand) for potential interactions with the immunological tumor network is not completely understood. This article provides a comprehensive immune phenotype profile of a TRAIL-knockout (TRAIL^−/−^) mouse model and should serve as a tool to study the functional relevance of TRAIL in proof-of-concept studies.

**Abstract:**

The TNF-superfamily member TRAIL is known to mediate selective apoptosis in tumor cells suggesting this protein as a potential antitumor drug target. However, initial successful pr-clinical results could not be translated into the clinic. Reasons for the ineffectiveness of TRAIL-targeting in tumor therapies could include acquired TRAIL resistance. A tumor cell acquires TRAIL resistance, for example, by upregulation of antiapoptotic proteins. In addition, TRAIL can also influence the immune system and thus, tumor growth. We were able to show in our previous work that TRAIL^−/−^ mice show improved survival in a mouse model of pancreatic carcinoma. Therefore, in this study we aimed to immunologically characterize the TRAIL^−/−^ mice. We observed no significant differences in the distribution of CD3^+^, CD4^+^, CD8^+^ T-cells, Tregs, and central memory CD4^+^ and CD8^+^ cells. However, we provide evidence for relevant differences in the distribution of effector memory T-cells and CD8^+^CD122^+^ cells but also in dendritic cells. Our findings suggest that T-lymphocytes of TRAIL^−/−^ mice proliferate at a lower rate, and that the administration of recombinant TRAIL significantly increases their proliferation, while regulatory T-cells (Tregs) from TRAIL^−/−^ mice are less suppressive. Regarding the dendritic cells, we found more type-2 conventional dendritic cells (DC2s) in the TRAIL^−/−^ mice. For the first time (to the best of our knowledge), we provide a comprehensive characterization of the immunological landscape of TRAIL-deficient mice. This will establish an experimental basis for future investigations of TRAIL-mediated immunology.

## 1. Introduction

The TNF-superfamily member TRAIL is known to mediate selective apoptosis in tumor cells which, together with a nearly nonexistent systemic toxicity, suggests this molecule as a potential antitumor drug [1]. However, initial successful pre-clinical results could not be translated into the clinic [2]. Reasons for the ineffectiveness of recombinant TRAIL or TRAIL agonists in the clinic are that tumor cells can become resistant to TRAIL-induced apoptosis, and the characterization of the underlying mechanisms is subject of ongoing research. Another currently less considered mechanism are the effects of TRAIL on the immunological microenvironment of the tumor.

In humans, two TRAIL receptors are known to have a functional death domain and thus can induce apoptosis: TRAIL receptor 1 (DR4) and TRAIL receptor 2 (DR5). In mice, only one TRAIL receptor with a functional death domain exists, namely TRAIL receptor 2 (DR5), which, however, is structurally not homologous to the two human DRs. TRAIL can also bind to two antiapoptotic decoy receptors (DcRs), DcR1 and DcR2. Indeed, overexpression of corresponding decoy receptors results in an inhibition of TRAIL-induced apoptosis [3].

Apart from signal transduction pathways resulting in apoptosis or necroptosis, TRAIL can activate numerous other signal transduction pathways including the NFkB, MAPK, Src, and PI3K pathways, which are known to maintain carcinoma cells [4]. In this context it has been demonstrated that with an appropriate stimulation under certain conditions, TRAIL can promote immune cell migration and invasion into tumors [5]. On the other hand, stimulation with TRAIL can lead to the release of cytokines by tumor cells, which in turn affects the immune system [6]. 

When studying the pleiotropic effect of TRAIL on cells of the immune system, T-lymphocytes became the focus of interest early on. Previous studies suggest that TRAIL acts on the T-cell milieu and thus, influences the adaptive immune response. The inhibition of T-cell-activation through apoptosis-independent pathways has been demonstrated in multiple experimental autoimmune disease models [7,8,9,10]. With regard to interactions of recombinant TRAIL with CD4^+^ cells, Lehnert et al. showed that in a culture of human CD4^+^ cells, TRAIL inhibits the proliferation of CD3/CD28-activated CD4^+^ cells without inducing apoptosis [11].

Dendritic cells (DCs) are a key interface between the innate and adaptive immune system [12]. The sensitivity of DCs towards TRAIL-induced apoptosis and their interaction with TRAIL in the tumor microenvironment are not yet fully understood. For future investigation of possible cancer therapies in a mouse model, the profiling of the immunological tumor microenvironment should be also expanded on cells of the innate immune system. 

To our knowledge, there is no published record of a comprehensive immune phenotype profile of TRAIL in a mouse model. As further potential interactions of TRAIL with the immunological network tumors have still to be examined, a comprehensive phenotyping of TRAIL in a mouse model will shed new light into TRAIL-mediated immunology.

## 2. Materials and Methods

### 2.1. Mice

8- to 12-week-old naïve wildtype C57BL/6 mice with body weights of 20–25 g were obtained from Charles River Laboratories (Sulzfeld, Germany) and were allowed to adapt to the new environment for about 14 days. TRAIL-knockout (TRAIL^−/−^) mice on C57BL/6 background were obtained from Amgen (Seattle, WA, USA). TRAIL deficiency was confirmed by the polymerase chain reaction of genomic DNA from ear biopsies, using primer pairs specific to the TRAIL-knockout allele (5’ gCC CTg AAT gAA CTg CAg gAC G 3’ and 3‘ CAC ggg TAg CCA ACg CTA TgT C5’) and WT TRAIL allele (5’ AAA gAC ggA TgA ggA TTT CTg gg 3’ and 3’ gAC AgA ACA CCA TAT TgC Tgg Cg 5’). Mice received food and water ad libitum. Animal rooms had a 12:12-h circadian cycle and were maintained at constant temperature and humidity. All animal studies had been approved by the ethics committee for animal care of the Landesamt für Gesundheit und Soziales Berlin. The experiments were done according to the German Animal Welfare Act (TierSchG).

### 2.2. Cell Isolation and Culture

Mice were sacrificed by cervical dislocation under deep anesthesia. Spleen, kidney, liver, small intestine, and lymph nodes were extracted. Spleen and lymph nodes were separately passed through a 100-μm nylon mesh (BD falcon cell strainer; BD Bioscience) and washed with full medium, containing RPMI 1640 media (Corning), and supplemented with 10% fetal bovine serum (FBS, Gibco) and 1% Penicillin/Streptomycin (PAN Biotech). Lymphocytes were isolated from the cell suspension using histopaque (Sigma-Aldrich) and filtered through a 40 µm nylon mesh. Cells were stored on ice with 2% bovine serum albumin (BSA, Biochrom) in phosphate-buffered saline (PBS). 

The small intestines were incubated in extraction media containing RPMI, EDTA, FBS, and dithiothreitol (DTT)-Solution for 15 min at 37 °C and 200 rpm. Afterwards, the intestine tissue was digested in RPMI 1640 media, Collagenase IVa (2 mg/mL, Roche), and DNase I (40 U/mL, Roche) for 40 min at 37 °C and 200 rpm. For cell extraction from the liver and kidney tissue, the same digestion was performed. The reaction was stopped by adding 10 mL ice-cold media RPMI 1640. The subsequent steps for lymphocyte isolation were performed as described above.

Additionally, for the extraction of lymphocytes from the kidney, CD45 beads were added to the cell suspension and magnetically labeled cells were purified over MACS^®^ LS columns (Miltenyi Biotec, Auburn, CA, USA) according to the manufacturer’s instructions.

If incubation was needed, cells were cultured in a conventional environment at 37 °C, 5% CO_2_ and 100% humid atmosphere.

### 2.3. Flow Cytometry

Cells were analyzed by fluorescence-activated cell sorting (FACS) on BD LSRFortessa X-20 (BD Biosciences) to reveal their immune phenotype within the cell populations of interest. For surface staining, after blocking of the samples with FcBlock (Miltenyi Biotec, Auburn, CA, USA) the cells were stained according to the manufacturer’s instructions with the antibodies listed in Table 1 and Table 2 for DCs. Unwanted cell populations were intentionally excluded from the flow cytometry analysis using a “dump channel” for the dead cells (fixable Live/dead, BV510) as well as the murine pan B-cell marker B220^+^ (BV510). FACS data were analyzed using FlowJo software v10 (Treestar Inc., Ashland, OR, USA) with polyfunctionality of cells being assessed using hierarchical gating.

For intracellular staining, cells were fixed with FoxP3 Fix/Perm Buffer (eBiosciences) for 30 min at 4 °C, permeabilized with FoxP3 Perm Buffer (eBiosciences), and then stained with AF647-conjugated FoxP3 and PE-eFluor 610-conjugated Ki67 antibody. All intracellular staining procedures were performed after surface staining. 

The gating strategy for the identification of the different immune cell subsets within the examined organs is depicted in the supplementary figures. 

### 2.4. Proliferation Assay of CD3^+^ Cells 

CD3^+^ T-cells were purified from the spleen of wildtype and TRAIL^−/−^ mice using the MACS^®^ cell-sorting system (Pan T cell isolation kit II, Miltenyi Biotec, Auburn, CA, USA) according to the manufacturer’s instructions, specifically using an antibody cocktail (provided by the manufacturer) against CD11b, CD11c, CD19, CD45R (B220), CD49b (DX5), CD105, Anti-MHC-class II, and Ter-119 over MACS LD columns. Resulting cells were >95% CD3 positive by FACS analysis. 96-well plates were incubated over at least one hour at 37 °C with an anti-CD3 antibody at a concentration of 1 µg/mL and afterwards washed three times with PBS. CD3^+^ T-cells were stained with carboxyfluorescein succinimidyl ester (CFSE, LifeTechnologies, Invitrogen) at a concentration of 4 µM and 5 × 10^4^ cells per well in the coated 96-well plate and distributed in 200 µL. Furthermore, an anti-CD28 antibody (BioLegend) at a concentration of 1 µg/mL was added. The cells were then treated with recombinant soluble murine TRAIL (Biolmol GmbH, Hamburg, Germany) (purity 95%, <1.0 EU per 1 g) and dissolved in sterile RPMI-1640 medium in different concentrations (0, 100 and 1000 ng/µL final concentration). Proliferation was checked by FACS on day 2 and 3.

### 2.5. Suppression Assay

CD4^+^CD25^−^ effector T-cells (Teff) and CD4^+^CD25^+^ regulatory T-cells (Treg) were purified from the spleen of wildtype and TRAIL^−/−^ mice using the MACS^®^ cell-sorting system (CD4^+^CD25^+^ Regulatory T Cell Isolation Kit, mouse in combination with LD columns) according to the manufacturer’s instructions. Specifically, all non-CD4^+^ are labeled with an antibody cocktail against CD8, CD11b, CD45R, CD49b, Ter-119, and Anti-Biotin, and depleted. In a second step, for the positive selection of Tregs, the CD4^+^ cells were incubated with CD25^+^ MicroBeads. To confirm the Teff and Treg phenotype, the cells were first labeled with the cell surface antibodies CD4-PECy7 and CD25-APC and then fixed and permeabilized for an intracellular staining as described above. CD4^+^CD25^−^FOXP3^−^ and CD4^+^CD25^+^FOXP3^+^ subsets were confirmed to be >95% pure by FACS analysis. CD4^+^CD25^+^FOXP3^+^ regulatory T-cells were stimulated overnight (18 h) in IL-2-enriched medium (200 U/mL murine IL2). Meanwhile, CD4^+^CD25^−^ effector cells were cultured in an incubator in RPMI-1640 medium without stimulating agents. The next day, 96-well plates were coated over one hour at 37 °C with an anti-CD3 antibody at a concentration of 1 µg/mL and afterwards washed three times with PBS. CD4^+^CD25^−^ T-cells were stained with CFSE and distributed in the coated 96-well plate, followed by an anti-CD28 antibody at a concentration of 1 µg/mL. Proliferation and suppression were checked by FACS on day 3.

### 2.6. Statistical Methods

Graphs were generated and statistical analysis was performed using GraphPad Prism v9.5.0 (GraphPad, La Jolla, CA, USA). After testing for normal distribution, ANOVA (with Tukey post hoc test) was chosen for multiple comparisons. For 2-group comparisons, 2-tailed, unpaired student’s *t*-test or Mann-Whitney U test were used depending on the values distribution. A *p* value < 0.05 was considered significant in all statistical tests. All bar graphs show mean ± SEM.

## 3. Results

### 3.1. Immune Phenotyping of TRAIL^−/−^ Mice

To assess the immunological profile of TRAIL^−/−^ mice and their comparison to wildtype C57BL/6 mice, we isolated leukocytes from multiple lymphoid (spleen, lymph nodes, small intestines) and non-lymphoid tissues (liver and kidney) and analyzed them by FACS.

#### Frequency of Dendritic Cell (DC) Populations in a TRAIL^−/−^ Mice

**Figure 1:**
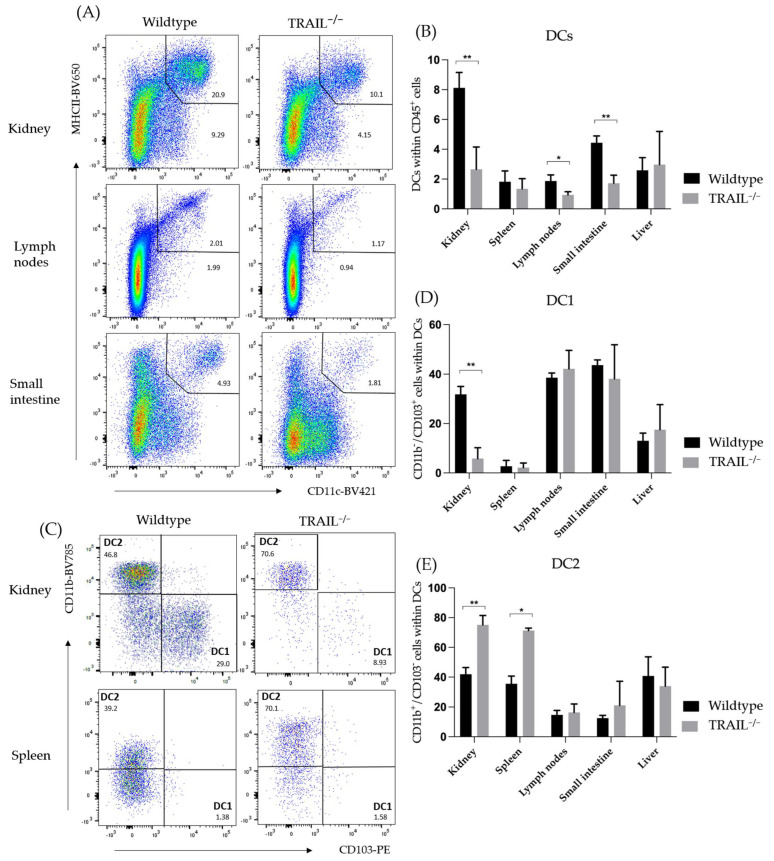
Distribution of DCs in the kidney, spleen, lymph nodes, liver, and small intestines of TRAIL^−/−^ mice *versus* wildtype C57BL/6 mice. (**A**) Exemplary dot plots illustrate higher frequencies of CD11c^+^MHCII^+^ dendritic cells in the kidney, lymph nodes, and small intestine of a TRAIL^−/−^ mice. (**C**) Exemplary dot plots illustrate distribution of CD103^+^MHCII^+^ DC1 and CD11b^+^MHCII^+^ DC2 subtypes in kidney and spleen of TRAIL^−/−^ mice *versus* wildtype C57BL/6 mice. (**B**) Histogram compares means ± SEM of all CD11c^+^MHCII^+^ DCs in the different organs (*n* = 6). (**D**,**E**) Exemplary dot plot illustrate the distribution of CD103^+^MHCII^+^ DC1 and CD11b^+^MHCII^+^ DC2 subtypes in kidney, spleen, lymph nodes, small intestine, and liver ((**B**,**D**,**E**) *t*-test, *n* = 6 per group, * *p* < 0.05, ** *p* < 0.01).

DCs are critical regulators of the primary immune response due to their ability to stimulate the activation and differentiation of naïve T-cells. Legge et al. classified dendritic cells into CD8 negative (DC2) and CD8 positive (DC1) DCs and assigned characteristic cytokines to each, namely IL-10 and IL-12, respectively. CD8^-^ DCs directly inhibit T-cell function by changing the CD28 expression and, on the other hand, indirectly through downregulation of IL-12 from CD8^+^ DCs by negative regulation of IL-10 [13]. Of special interest are CD103^+^ DC2s, which are typically sparsely represented but play a key role in the antigen cross-presentation as well as in stimulating the generation of CD4^+^CD25^+^FoxP3^+^ Tregs [14]. We found significantly decreased frequencies of overall CD11c^+^MHCII^+^ DCs in the kidney, lymph nodes, and small intestines in TRAIL^−/−^ mice (Figure 1). After investigating the distribution of the DC subtypes 1 and 2, we additionally found significantly reduced frequencies of CD103^+^MHCII^+^ DC1 cells in the kidney of TRAIL^−/−^ mice (* *p* < 0.01). In comparison, CD11b^+^MHCII^+^ DC2 subtypes were significantly more present in the kidney and spleen of TRAIL^−/−^ mice *versus* wildtype C57BL/6 mice (** *p* < 0.01 resp. * *p* < 0.05). The gating strategy to identify DCs is depicted in Supplemental Figure S1.

**Figure 2:**
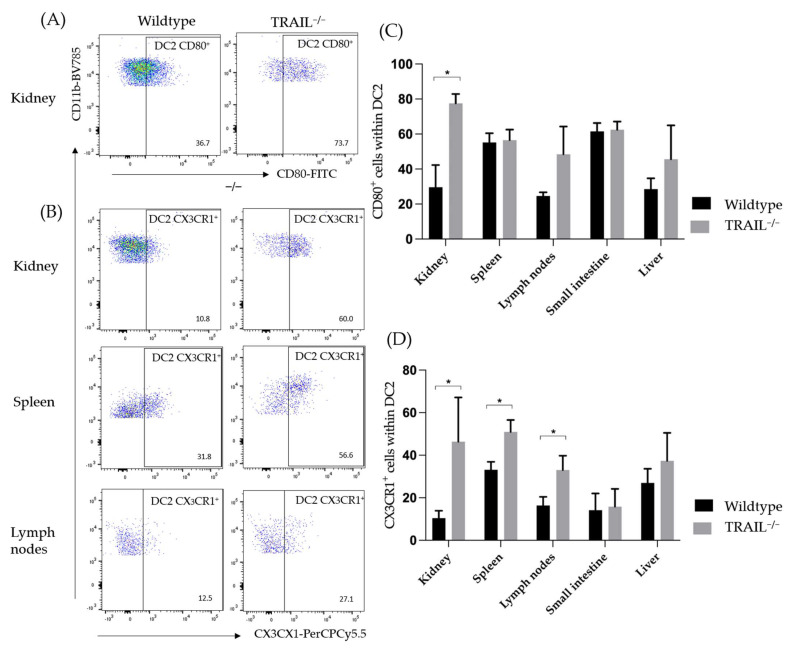
Distribution of CD80^+^ (**A**) and CX_3_CR1^+^ (**B**) CD11c^+^MHCII^+^ DCs in the kidney, spleen, lymph nodes, small intestines, and liver of TRAIL^−/−^ mice *versus* wildtype C57BL/6 mice. (**A**,**B**) Exemplary dot plot illustrate the CD80 (**A**) and CX_3_CR1 (**B**) expression on CD11c^+^MHCII^+^ dendritic cells in the kidney, spleen, and lymph nodes of a TRAIL^−/−^ mice. (**C**,**D**) Histogram compares means ± SEM of all CD80^+^ (**C**), CX_3_CR1^+^ and (**D**) CD11c^+^MHCII^+^ DCs in kidney, spleen, lymph nodes, intestine, and liver ((**C**,**D**) *t*-test, *n* = 6 per group, * *p* < 0.05).

CD80, primarily expressed on antigen-presenting cells, and mostly DCs, is known to trigger T-cell activation and to promote NK-cell-mediated cytotoxicity [15,16]. In the context of the tumor microenvironment, reduction of CD80-expression in cancer cells is a powerful mechanism of immune response evasion [17,18]. We found a pronounced CD80-expression of TRAIL^−/−^ CD11b^+^MHCII^+^ DC2s in the spleen compared to wildtype mice (Figure 2). The CX_3_C chemokine receptor 1 (CX_3_CR1) is widely expressed in T- and NK-cells, monocytes, macrophages, and most dendritic cells [19]. CX_3_CR1 promotes the recruitment and migration of immune cells to the inflammation origin. This axis of CX_3_CR1 with its ligand Fractalkine CX_3_CL1 is suggested to play an ambiguous role in oncogenesis since it can exercise pro- and anti-tumor functions [20]. Previous studies have further demonstrated that CX_3_CR1 may play a key role in the regulation of immune tolerance and inflammation through DCs [21]. Therefore, we interrogated the frequencies of CX_3_CR1^+^ CD11b^+^MHCII^+^ DC2 cells of the lymphatic organs of TRAIL^−/−^ mice. Intriguingly, we found a significantly increased number of CX_3_CR1^+^ CD11b^+^MHCII^+^ DC2s in the spleen, kidney, and lymph nodes TRAIL^−/−^ mice (* *p* < 0.05) (Figure 2).

**Figure 3:**
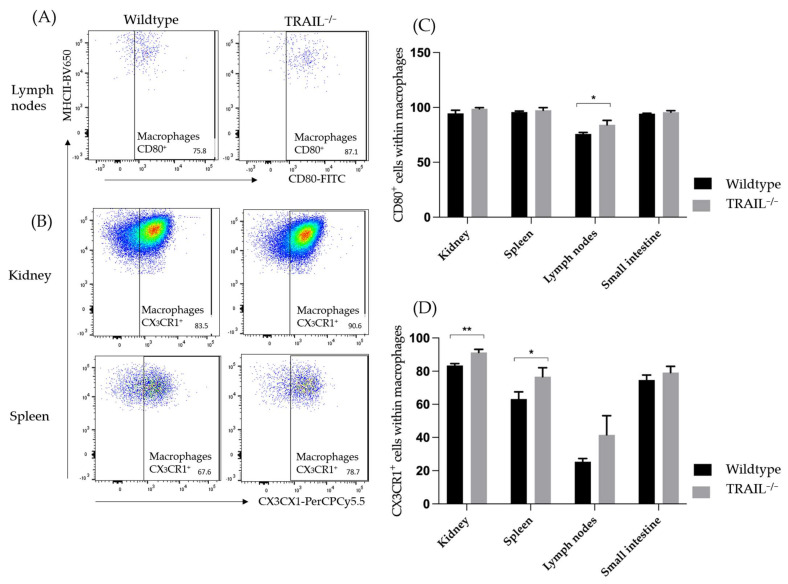
Distribution of CD80^+^ (**A**) and CX_3_CR1^+^ (**B**) macrophages in the lymph nodes, kidney, and spleen of TRAIL^−/−^ mice *versus* wildtype C57BL/6 mice. (**A**,**B**) Exemplary dot plot illustrate the CD80 (**A**) and CX_3_CR1 (**B**) expression on macrophages in the kidney, spleen, and lymph nodes of TRAIL^−/−^ mice *versus* wildtype C57BL/6 mice. (**C**,**D**) Histogram compares means ± SEM of all CD80^+^ (**C**) and CX_3_CR1^+^ (**D**) macrophages in the kidney, spleen, lymph nodes, and small intestine ((**C**,**D**) Mann-Whitney test, *n* = 6 per group, * *p* < 0.05, ** *p* < 0.01).

We did not observe any significant differences in the overall distribution of macrophages between TRAIL^−/−^ and wildtype mice. We further investigated the expression of CD80 and CX_3_CR1 in macrophages. CD80 is a marker of classically polarized macrophages (M1) [22]. M1 macrophages typically produce pro-inflammatory cytokines such as IL-6, IL-12, and TNF-alpha. Overall, we found no increase of the CD80 expression on the macrophages except in the TRAIL^−/−^ lymph nodes (Figure 3). Previous studies suggest a role of CX_3_CR1 in tumor metastasis by promoting angiogenic macrophage survival and angiogenesis in the tumor microenvironment [23]. Increased frequencies of CX_3_CR1^+^ macrophages were found on macrophages in the murine TRAIL^−/−^ kidney and spleen (** *p* < 0.01 resp. * *p* < 0.05). The gating strategy to identify macrophages is depicted in Supplemental Figure S1.

**Figure 4:**
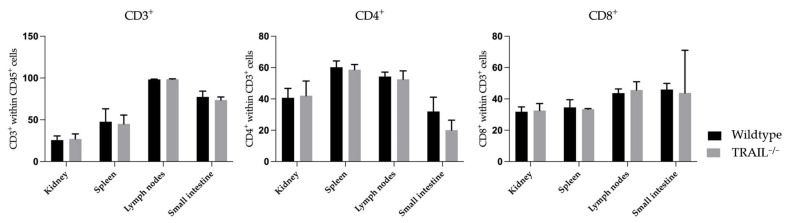
Histogram compares means of frequencies ± SEM of CD3, CD4, and CD8 T-cells in the kidney, spleen, lymph nodes, and small intestine of TRAIL^−/−^ mice *versus* wildtype C57BL/6 mice (*t*-test for CD3 and CD4, Mann-Whitney test for CD8, *n* = 6–7 per group).

No significant differences In the distribution of CD3^+^, CD4^+^, and CD8^+^ T-cells in the kidney, spleen, lymph nodes, and small intestines of TRAIL^−/−^ mice *versus* wildtype C57BL/6 mice were detected (Figure 4). The gating strategy to identify main lymphocyte subpopulations is depicted in Supplemental Figure S2.

**Figure 5:**
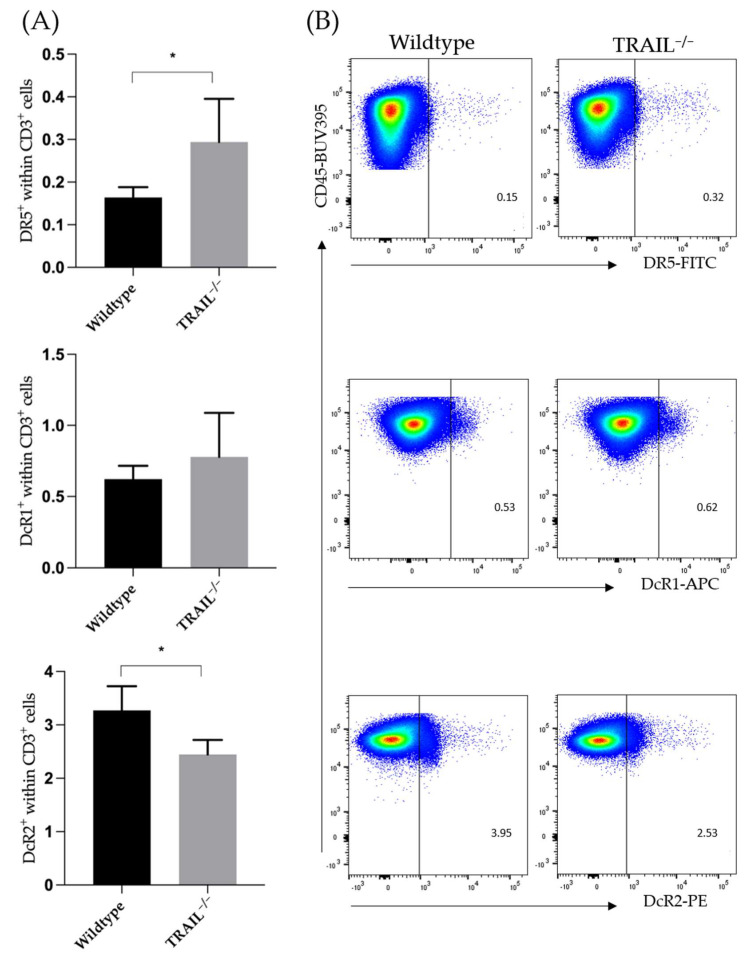
(**A**) Bar graph compares frequencies ± SEM of DR5, DcR1, and DcR2 expression on the CD3^+^ lymphocytes in the spleen of TRAIL^−/−^ mice *versus* wildtype C57BL/6 mice (*n* = 5). (**B**) Exemplary dot plots illustrate DR5, DcR1, and DcR2 expression (*t*-test, *n* = 5 per group, * *p* < 0.05).

In addition to the two TRAIL receptors that have a functional death domain and can thus, induce apoptosis (DR4 and DR5), other TRAIL receptors exist that act as decoy receptors. These include TRAIL receptor 3 (DcR1), which has no intracellular domain, and the functionally inactive TRAIL receptor 4 (DcR2) [24]. In this study, we found significantly increased frequencies of DR5 (* *p* < 0.05) on CD3-lymphocytes in the murine TRAIL^−/−^ spleen. In contrast, we observed a decreased expression of the functionally inactive DcR2 receptor in TRAIL^−/−^ (* *p* < 0.05) (Figure 5).

**Figure 6:**
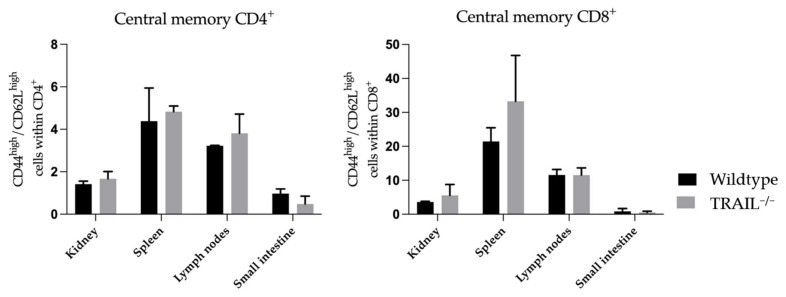
Histogram compares means ± SEM of CD4^+^ and CD8^+^ central memory T-lymphocytes in the kidney, spleen, lymph nodes, and small intestines of TRAIL^−/−^ mice *versus* wildtype C57BL/6 mice (*t*-test, *n* = 6 per group).

We observed no significant differences in the presence of central memory CD4^+^ and CD8^+^ cells (Figure 6). 

**Figure 7:**
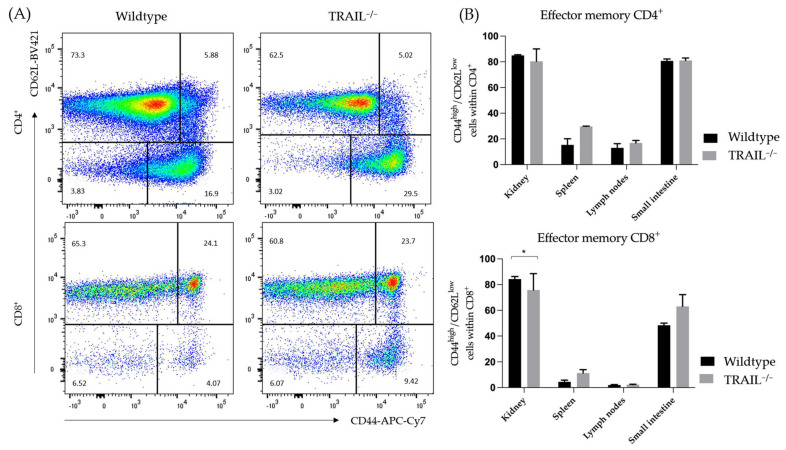
(**A**) Exemplary dot plot represents the presence of effector memory CD62^low^CD44^high^ cells within the CD4^+^ and CD8^+^ population in the spleen of TRAIL^−/−^ mice *versus* wildtype C57BL/6 mice. (**B**) Histogram compares means ± SEM of effector memory CD62^low^CD44^high^ cells within the CD4^+^ resp. CD8^+^ population in the spleen, lymph nodes, kidney, and small intestine of TRAIL^−/−^ mice *versus* wildtype C57BL/6 mice (*t*-test, *n* = 6 per group, * *p* < 0.05).

Next, we explored the distribution of CD4^+^ and CD8^+^ (CD62^low^CD44^high^) effector memory cells and found that these lymphocyte subtypes are more pronounced in the spleen in of TRAIL^−/−^ mice (Figure 7).

**Figure 8:**
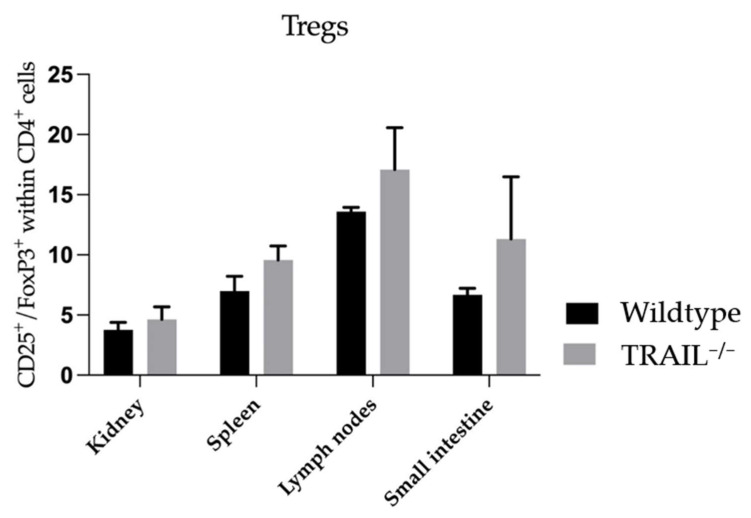
Histogram compares means ± SEM of Tregs in kidney, spleen, lymph nodes, and small intestines of TRAIL^−/−^ mice *versus* wildtype C57BL/6 mice (*t*-test, *n* = 6 per group).

Next, we examined the frequencies of Tregs in lymphatic and non-lymphatic organs. We observed a tendency towards accumulation of Tregs in the kidney, spleen, lymph nodes, and small intestines of TRAIL^−/−^ mice *versus* wildtype C57BL/6 mice; however, it was not significant (Figure 8).

**Figure 9:**
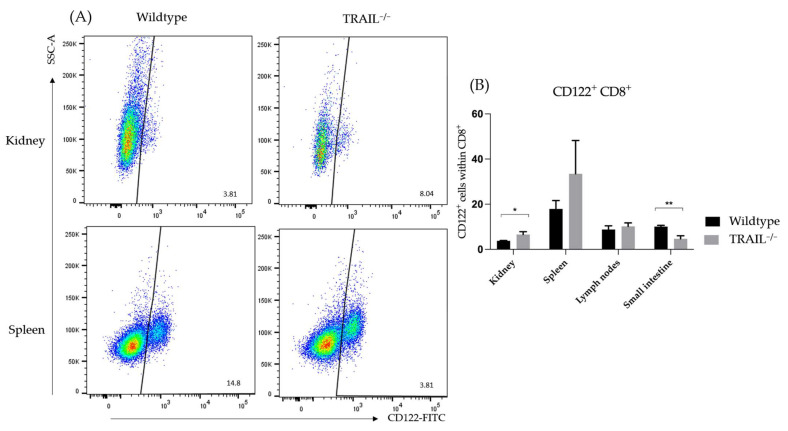
(**A**) Exemplary dot plot represents the presence of CD8^+^CD122^+^ T-cells in the kidney and small intestine of TRAIL^−/−^ *versus* wildtype mice. (**B**) Histogram compares means ± SEM of CD8^+^CD122^+^ T-cells in the spleen, lymph nodes, kidney, and small intestine of TRAIL^−/−^ mice *versus* wildtype C57BL/6 mice (*t*-test, *n* = 6 per group, * *p* < 0.05, ** *p* < 0.01).

CD8^+^CD122^+^ T-cells belong to a central-memory regulatory T-cell subset. They are known to exhibit potent suppressant functions towards auto- as well as alloimmunity [25]. We found an increased number of CD8^+^CD122^+^ T-cells in the kidney (* *p* < 0.05) and spleen (not significant, *p* = 0.157) of TRAIL^−/−^ mice. Interestingly, we detected a decreased presence of CD8^+^CD122^+^ T-cells in the TRAIL^−/−^ small intestine (Figure 9).

**Figure 10:**
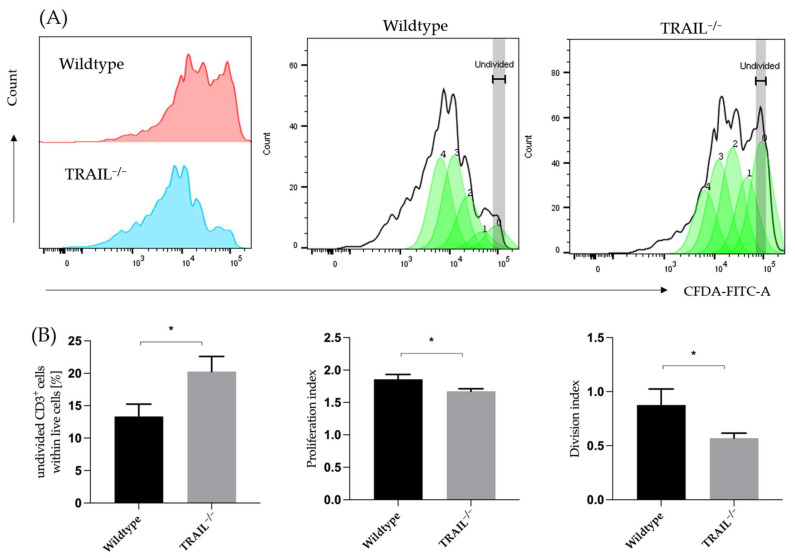
CD3^+^ T-cell proliferation in TRAIL^−/−^ and wildtype mice. (**A**) FACS histogram of proliferation peaks. (**B**) Cumulative percentage of undivided CD3^+^ T-cells, proliferation index, and division index of CD3^+^ T-cells from of TRAIL^−/−^ mice *versus* wildtype C57BL/6 mice (*t*-test, *n* = 6–8, * *p* < 0.05). Graphs show mean ± SD.

We analyzed the proliferation capacity of wildtype and TRAIL-deficient CD3^+^ lymphocytes. We isolated CD3^+^ lymphocytes from the splenocytes using MACS and verified the isolation purity via FACS. The proliferation was analyzed after 72 h of stimulation with CD3/CD28 using FACS. Our findings suggest that wildtype CD3^+^ lymphocytes proliferate at a significantly higher rate (*p* = 0.037) compared to the TRAIL-knockout lymphocytes (Figure 10). The gating strategy to identify CFSE-based proliferation of CD4^+^ T cells is depicted in Supplemental Figure S3.

**Figure 11:**
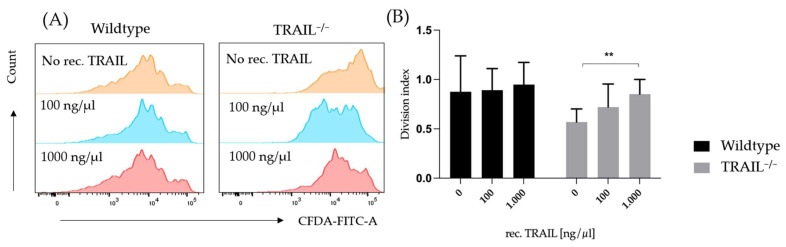
Effect of treatment with different concentrations of recombinant TRAIL (0, 100 and 1000 ng/µL) on CD3^+^ T-cell proliferation in TRAIL^−/−^ and wildtype mice. (**A**) FACS histogram of proliferation peaks. (**B**) Division index of CD3^+^ T-cells from TRAIL^−/−^ mice *versus* wildtype C57BL/6 mice after TRAIL treatment in different concentrations (*n* = 6–8 per group, two-way ANOVA, Tukey correction test, ** *p* < 0.01). Graphs show mean ± SD.

To examine the effect of exogenous TRAIL on lymphocyte proliferation, we treated CD3^+^ lymphocytes with different concentrations of recombinant murine TRAIL. The experiment was performed as described in 2.4. Recombinant TRAIL was added to the batches in a final concentration of 0, 100, and 1000 ng/µL. Proliferation was measured 72 h after treatment by FACS. The TRAIL treatment had a significant impact on the proliferation of TRAIL-deficient lymphocytes (** *p* < 0.01); however, it had no effect on the wildtype group (Figure 11).

**Figure 12:**
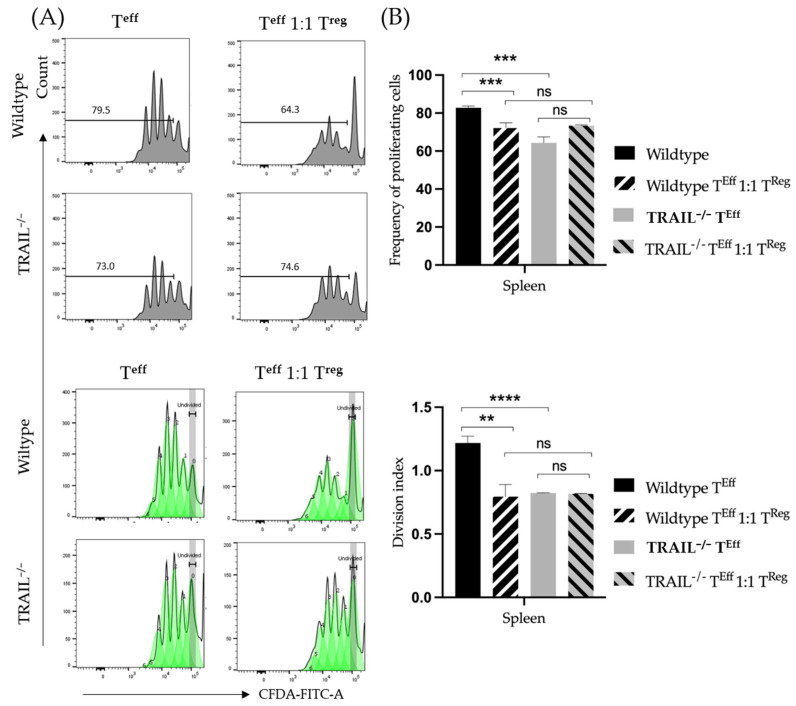
(**A**) CD4^+^CD25^+^ T-cell proliferation alone *versus* proliferation in the presence of CD4^+^CD25^+^ regulatory T-cells from TRAIL^−/−^ mice *versus* wildtype C57BL/6 mice. (**B**) Histogram compares the TRAIL effect on the suppressive capacity of the regulatory T-cells of TRAIL^−/−^ mice *versus* wildtype C57BL/6 mice. Data were obtained (*n* = 4–6 per group, one-way ANOVA, Tukey post-test, ** *p* < 0.01, *** *p* < 0.001, **** *p* < 0.0001). Graphs show mean ± SD.

Previous studies suggest that regulatory CD4^+^ cells exert suppressive properties on T-effector cells via the expression of TRAIL [26]. We hypothesized that the suppressive properties of TRAIL-deficient regulatory CD4^+^ cells differ from those of wildtype mice. We isolated CD4^+^CD25^+^ as well as CD4^+^CD25^−^ lymphocytes using MACS. We then performed a suppression assay as described in 2.5. We cultivated wildtype CD4^+^CD25^+^ lymphocytes with CD4^+^CD25^−^ lymphocytes as well as TRAIL-deficient CD4^+^CD25^+^ with CD4^+^CD25^−^ cells. The assay was performed in the absence of antigen-presenting cells. Proliferation and suppression rates were measured by FACS on day 3. As shown in Figure 12, wildtype regulatory T-cells exert significantly stronger suppression over the effector T-cell proliferation capacity (*p* = 0.0006), whereas in TRAIL^−/−^ mice the proliferation of effector T-cells was barely affected in the presence of CD4^+^CD25^+^ cells.

## 4. Discussion

The TNF-superfamily member TRAIL is known for its selective apoptosis induction in tumor cells through engagement of DR4 and DR5 [1,3]. Based on pre-clinical data, TRAIL appeared to be a promising anti-tumor target. However, it has failed to show positive outcomes in the clinic [2]. Besides, recombinant human TRAIL as an anti-cancer therapeutic, which was unsuccessful in clinical trials due to poor bioavailability and short half-life, agonistic TRAIL-receptor antibodies have also shown limited effectiveness. Reasons for this are possibly due to acquired resistance of primary tumor cells to DR4/5-induced signaling [27]. Regardless, a general immunosuppressive effect of TRAIL could also influence the tumor-specific immune response and thus, lead to tumor progression. TRAIL-deficient mice have been broadly implemented in pre-clinical studies as a model for TRAIL research in the context of oncology. TRAIL^−/−^ mice have been shown to be more susceptible to carcinogenic substances, are more vulnerable for metastases, and show predisposition of autoimmune diseases such as diabetes [1,27,28,29,30]. However, we have seen in preliminary work that TRAIL^−/−^ mice have a survival advantage in an orthotopic pancreatic carcinoma model in fully immunocompetent mice. In this model, the administration of recombinant TRAIL led to significantly increased tumor growth. TRAIL^−/−^ mice, on the other hand, had significantly smaller tumors and showed a highly significantly improved survival compared to corresponding wildtype mice. We have provided evidence that the proportion of regulatory CD4^+^ cells in pancreatic tumors of TRAIL^−/−^ mice is significantly lower compared to wildtype mice. TRAIL treatment in wildtype mice, on the other hand, leads to a significantly increased proportion of regulatory CD4^+^ cells in the tumors while the proportion in the lymphoid organs does not change significantly [31]. These results suggest that not only the direct effect of TRAIL and corresponding agonistic receptor antibodies on tumor cells but also the interactions of these substances with the immune system can limit clinical use of TRAIL and TRAIL-agonists. In this regard, we have hypothesized that this survival benefit is caused by TRAIL’s interactions with the immune system. For this purpose, we have characterized the TRAIL^−/−^ mouse immunologically. 

In this study, we have found differences in distribution of T-cells and in DCs. We observed no significant differences in the distribution of CD3^+^, CD4^+^, CD8^+^ T-cells, Tregs, and central memory CD4^+^ and CD8^+^ cells in the lymphatic and non-lymphatic organs of TRAIL^−/−^ versus wildtype C57BL/6 mice. However, we found an increased number of CD8^+^CD122^+^ T-cells in the kidney and spleen of a TRAIL^−/−^ mouse and a decreased number of CD8^+^CD122^+^ T-cells in the TRAIL^−/−^ small intestine. CD8^+^CD122^+^ T-cells (CD44^high^CD62L^high^) are central memory T-cells with regulatory functions exerted through secretion of IL-10, TGFβ1 and IFNγ [25]. There is evidence that CD8^+^CD122^+^ T-cells can suppress allo- and autoimmunity in various murine disease models [32]. These cells do not express FoxP3; thus, they differ from the classical suppressor CD8^+^FoxP3^+^ subset [33]. Furthermore, we found an increased proportion of CD62^low^ CD44^high^ effector memory cells within the CD4^+^ and CD8^+^ populations in the TRAIL^−/−^ spleen. CD8^+^ effector memory cells are known to play an inferior role in antitumor immunity when compared to CD8^+^ central memory cells, based on their proliferative capacity [34]. 

The overall T-cell differences are inconclusive since TRAIL appears to promote both CD4 cell proliferation and suppression by Tregs. It has been previously shown that TRAIL suppresses the activation of T-cells [10]. Reports regarding the effects of TRAIL on regulatory and effector T-cells have been discordant. Ikeda et al. provided evidence that TRAIL promotes the proliferation of regulatory T-cells and inhibits the proliferation of Th1-cells, thereby suppressing autoimmunity [26]. It is assumed that these different effects are due to the expression of different TRAIL-receptors on the cell subpopulations: even though both subtypes express DR5-receptor, the regulatory T-cells also express mDcR1. In this study, we explored a different aspect, and our findings suggest that TRAIL is required for the proliferation of CD3^+^ T-cells, since first, lymphocytes from a TRAIL^−/−^ mouse proliferate at a significantly lower rate, and second, treatment with recombinant TRAIL can increase the proliferation of CD3^+^ T-cells in a dose-dependent manner. In this study, we have observed that TRAIL-deficient regulatory CD4^+^CD25^+^FoxP3^+^ T-cells are unable to unfold their suppressive capacity over the proliferation of the effector CD4^+^CD25^-^FoxP3^−^ T-cells. Unfortunately, in our study the murine regulatory T-cells have presented as fairly unstable and apoptosis could not be delayed for the purpose of further experiments, such as exploring the effects on suppression and proliferation after treatment with recombinant TRAIL. Therefore, further investigation is needed.

We have found decreased frequencies of overall CD11c^+^MHCII^+^ DCs in the kidney, lymph nodes, and small intestines of the TRAIL^−/−^ mouse. Specifically, there were reduced frequencies of CD103^+^MHCII^+^ DC1 cells in the kidney of TRAIL^−/−^ mice; however, we have observed increased frequencies of CD11b^+^MHCII^+^ DC2 cells in the kidney and spleen of TRAIL^−/−^ versus wildtype C57BL/6 mice. This is interesting because DC2s have different roles in tumor growth. Specifically in tumors that are less immunogenic (such as pancreatic carcinoma), DC2s seem to exert anti-tumorigenic effects [35]. This could provide a possible explanation for the better survival of TRAIL^−/−^ mice in the pancreatic carcinoma model.

In lung cancer, for example, TRAIL plays a major role in the polarization of macrophages [36]. Specifically, TRAIL can re-educate tumor-associated macrophages (TAMs) to an M1-like phenotype and induce cytotoxic effects in the tumor cells [36,37,38]. Furthermore, it has been shown that M1-macrophages promote TRAIL expression and as a result suppress tumor development and progression [39]. In a murine tumor model, myeloid-derived suppressor cells (MDSCs) show an increased expression of TRAIL receptors and render them more susceptible to TRAIL-induced apoptosis [40]. Dendritic cells and M2-macrophages also appear to be sensitive to TRAIL-induced apoptosis under certain circumstances [41,42]. Aside from the TRAIL^−/−^ lymph nodes, no significant increase of the CD80 expression on the macrophages was observed. Furthermore, we have found that macrophages and dendritic cells in TRAIL^−/−^ lymphatic organs express higher levels of CX_3_CR1. It has been shown that higher expression of CX_3_CR1 in macrophages correlates with higher metastasizing potential and poorer clinical prognosis in tumor patients [23]. 

To this day, there is no standardized protocol for immune phenotype profiling in a murine model in health and disease. New markers for defining a specific immune cell subset are constantly emerging. TRAIL remains an interesting focal point of investigations in an oncogenic and immunological sense, and the TRAIL^−/−^ mouse is a very useful tool in this context. Therefore, there is an urgent need for a comprehensive understanding of this experimental model for the purpose of exploring TRAIL interactions with the tumor microenvironment. 

## 5. Conclusions

We present for the first time a comprehensive immunological phenotype profile of a TRAIL^−/−^ mouse by systematically investigating the distributions of immune cell subsets in multiple lymphatic organs. Our findings suggest that the TRAIL^−/−^ T-lymphocytes proliferate at a lower rate, whereby the administration of recombinant TRAIL increases their proliferation. Furthermore, our study suggests that TRAIL is required for the suppression of the CD4^+^CD25^−^ effector T-cells (Teffs) by the CD4^+^CD25^+^ regulatory T cells (Tregs). These findings support the hypothesis that TRAIL interacts with the immunological milieu and therefore, can modulate tumor growth. 

## Figures and Tables

**Table 1:** List of the anti-mouse conjugated antibodies for immune cell analysis via FACS.

Marker	Fluorophore	Company
CD3	PerCP	Miltenyi Biotec GmbH, Bergisch Gladbach, Germany
CD8	BV605	BioLegend, San Diego, CA, USA
CD44	APC-eFluor780	eBioScience, Waltham, MA, USA
CD62L	BV421	BioLegend, San Diego, CA, USA
FoxP3	Alexa-fluor-647	BD Biosciences, San Jose, CA, USA
CD25	PE	Miltenyi Biotec GmbH, Bergisch Gladbach, Germany
CD45	BUV395	BD Biosciences, San Jose, CA, USA
CD122	FITC	BioLegend, San Diego, CA, USA
CD127	BV785	BioLegend, San Diego, CA, USA
DR5	VioBrightB515 (FITC)	Miltenyi Biotec GmbH, Bergisch Gladbach, Germany
Dcr1	APC	Miltenyi Biotec GmbH, Bergisch Gladbach, Germany
DcR2	PE	BioLegend, San Diego, CA, USA
Ki67	PE-eFluor 610	ThermoFisher, Waltham, MA, USA
B220 *	BV510	BioLegend, San Diego, CA, USA
Live/dead *	BV510	BioLegend, San Diego, CA, USA
CD4	PeCy7	BD Biosciences, San Jose, CA, USA
	BV711	BioLegend, San Diego, CA, USA

* “Dump channel”.

**Table 2:** List of the anti-mouse conjugated antibodies for the analysis of dendritic cells via FACS.

Marker	Fluorophore	Company
CD3 *	BV510	BioLegend, San Diego, CA, USA
NKp60 *	BV510	BioLegend, San Diego, CA, USA
B220 *	BV510	BioLegend, San Diego, CA, USA
CD45	UV395	BD Biosciences, San Jose, CA, USA
CD11c	BV421	BioLegend, San Diego, CA, USA
CD11b	BV785	BioLegend, San Diego, CA, USA
MHCII	BV650	BioLegend, San Diego, CA, USA
F4/80	APCCy7	BioLegend, San Diego, CA, USA
CD64	Pe-Cy7	BioLegend, San Diego, CA, USA
Ly6C	PE-Dazzle	BioLegend, San Diego, CA, USA
CD80	FITC	BioLegend, San Diego, CA, USA
CD8a	BV605	BioLegend, San Diego, CA, USA
CD103	PE	BioLegend, San Diego, CA, USA
CX3CR1	PerCPCy5.5	BioLegend, San Diego, CA, USA
CD38	BV711	BD Biosciences, San Jose, CA, USA
MHCI H2KD	Alexa700	BioLegend, San Diego, CA, USA
Live/dead *	BV510	BioLegend, San Diego, CA, USA

* “Dump channel”.

## Data Availability

A dataset of the present study can be requested from the corresponding author on reasonable request.

## Supplementary Materials

The following supporting information can be downloaded at: https://www.mdpi.com/article/10.3390/cancers15051475/s1, Figure S1: Gating strategy for identifying macrophages and dendritic cells. Figure S2: Gating strategy for identifying main lymphocyte subpopulations. Figure S3: Gating strategy for CFSE-based proliferation of CD4+ T cells.
