# Supplementary material for: Immune Phenotypic Characterization of a TRAIL-Knockout Mouse
Source: Cancers (Basel). 2023 Feb 25;15(5):1475. doi: 10.3390/cancers15051475 (PMC10000729; doi:10.3390/cancers15051475)
Supplement: Supplementary file 1 [file cancers-15-01475-s001.zip › cancers-2203346-supplementary.pdf]

Supplementary Figure 1 Stoyanova et al.

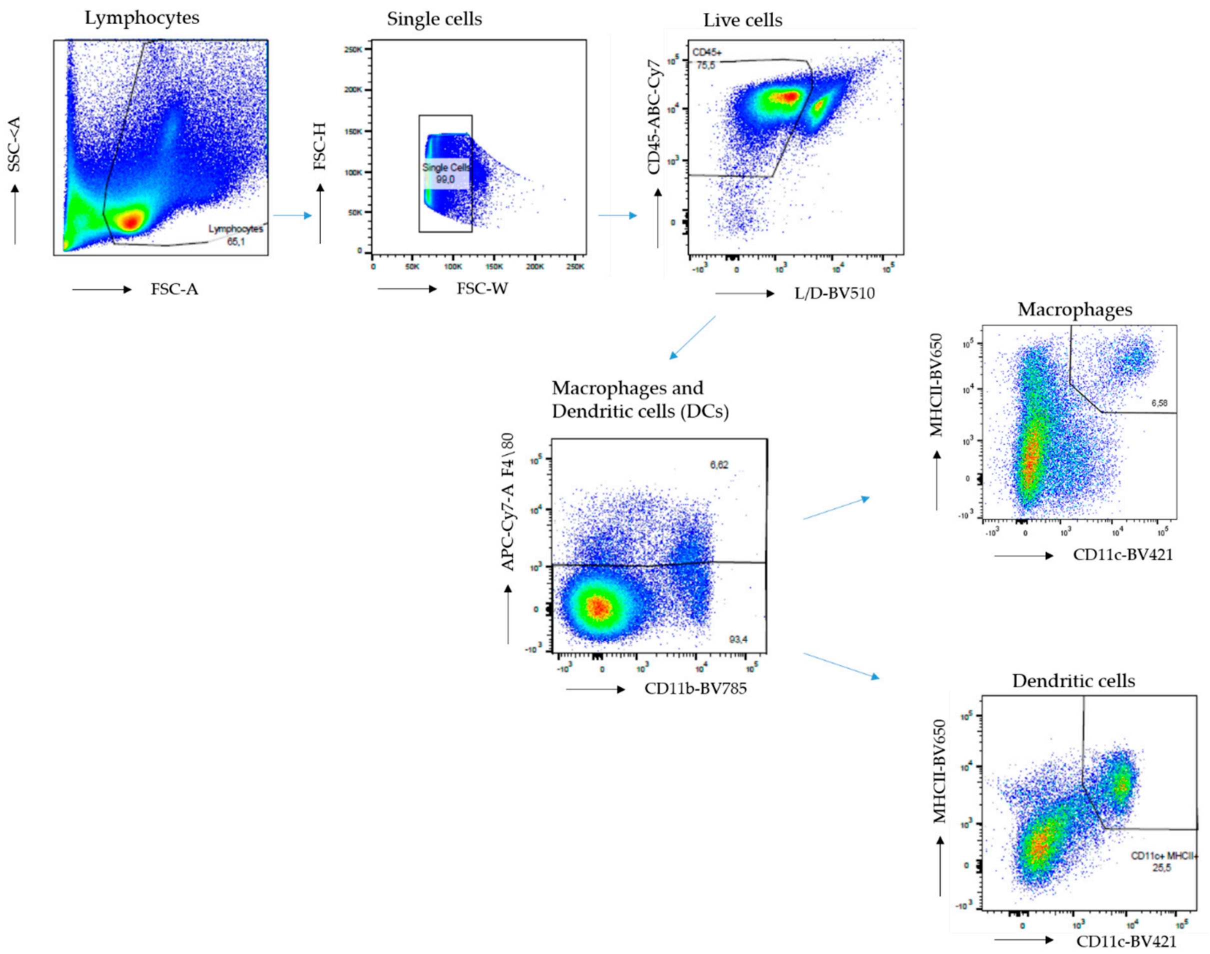

Gating strategy for identifying macrophages and dendritic cells. Lymphocytes were selected from a forward scatter area vs side scatter area dot plot, and single cells were subsequently selected in a forward scatter-area vs forward scatter height dot plot. Live CD45^{+} cells were identified and divided into DCs and macrophages.

Supplementary Figure 2 Stoyanova et al.

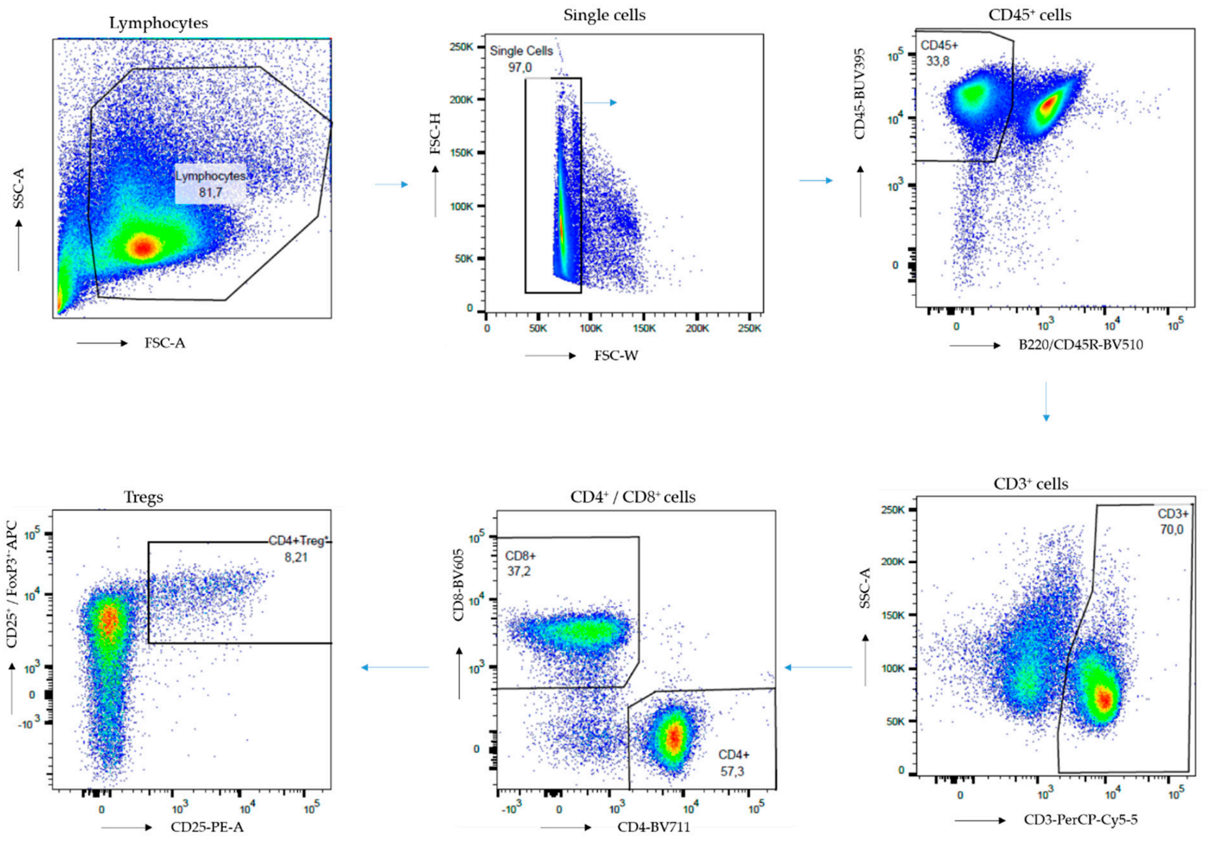

Gating strategy for identifying main lymphocyte subpopulations. Lymphocytes were selected from a forward scatter area vs side scatter area dot plot, and single cells were subsequently selected in a forward scatter-area vs forward scatter height dot plot. Live $CD45^+$ cells were subdivided from the B220-expressing B-lymphocytes. $CD3^+$, following $CD4^+$ and $CD8^+$cells were identified via co-staining with BUV395, BV711 and BV605 resp. Tregs were defined as $CD25^+$ $FoxP3^+$ cells.

Supplementary Figure 3 Stoyanova et al.

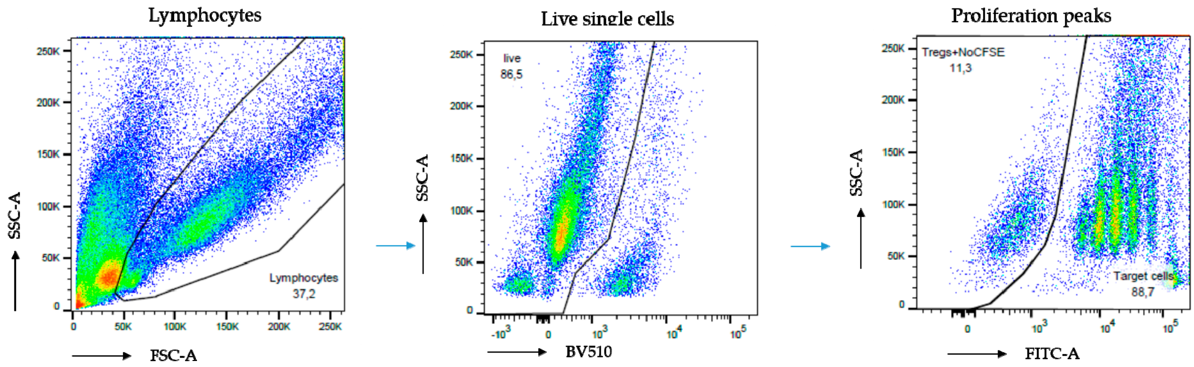

Gating strategy for CFSE-based proliferation of CD4⁺ T cells. Lymphocytes were selected from a forward scatter area vs side scatter area dot plot, and single cells were subsequently selected in a forward scatter-area vs forward scatter height dot plot. Live CD4⁺ cells were identified via staining with BV510. Proliferation peaks of Teffs were identified via CFSE-staining.
